# An Overview of Geometrical Optics Restricted Quantum Key Distribution

**DOI:** 10.3390/e23081003

**Published:** 2021-07-31

**Authors:** Ziwen Pan, Ivan B. Djordjevic

**Affiliations:** Department of Electrical & Computer Engineering, College of Engineering, The University of Arizona, 1230 E Speedway Blvd, Tucson, AZ 85721, USA; ivan@email.arizona.edu

**Keywords:** quantum key distribution, satellite, free-space channel

## Abstract

Quantum key distribution (QKD) assures the theoretical information security from the physical layer by safely distributing true random numbers to the communication parties as secret keys while assuming an omnipotent eavesdropper (Eve). In recent years, with the growing applications of QKD in realistic channels such as satellite-based free-space communications, certain conditions such as the unlimited power collection ability of Eve become too strict for security analysis. Thus, in this invited paper, we give a brief overview of the quantum key distribution with a geometrical optics restricted power collection ability of Eve with its potential applications.

## 1. Introduction

There has been a long history of cryptography [1,2,3,4,5,6]. Before the 20th century, cryptography was considered as an art that mainly relies on personal skills to construct or break codes, without proper theoretical study [7]. Focused on message confidentiality, classical cryptography was known to ensure secrecy in communications under different situations such as military or diplomat use or between spies. An important representative of classic cryptography is transposition ciphers, which rearrange the message to hide the original meanings. After the early 20th century, following the establishment of the information theory by Harry Nyquist, Ralph Hartley, and Claude Shannon [8,9,10,11,12,13], the study of cryptography started to exploit the tools of mathematics. Cryptography also became a branch of engineering, especially after the use of computers, which allows binary encryption of data. Two major schemes of modern cryptography include symmetric (private-key) cryptography, e.g., the Data Encryption Standard (DES) [14] and Advanced Encryption Standard (AES) [15], and asymmetric (public-key) cryptography, e.g., RSA algorithm [16]. Symmetric cryptography relies on the shared key between the communication parties (Alice and Bob), whereas in asymmetric cryptography, the encryption keys are different from decryption keys. In general, symmetric cryptography is more efficient than asymmetric cryptography with more concise designs, but it has difficulties when it comes to the safe distribution of the shared keys. On the other hand, asymmetric cryptography using a public key and a private key for encryption and decryption, respectively, relies upon mathematical problems termed one-way functions that are computationally infeasible from one direction (public key) [17], and are more widely used today for avoiding the risky stage of safe distribution of keys in symmetric cryptography.

However, with the fast development of quantum computing [18] and its potential in solving conventional one-way functions, it is possible to break the current encryption systems [19] with algorithms such as Shor’s algorithm [20] and Grover’s algorithm [21]; thus, the QKD is now becoming more and more important in the new era of information security. Different from the asymmetric cryptography used today, QKD is based on symmetric cryptography, guaranteeing the secure distribution of the secret keys with the laws of quantum mechanics that the measurement process generally disturbs the measured system. This can be used to detect eavesdropping actions as any adversaries would have to perform measurement to eavesdrop. Since the study of the first QKD protocol BB84 [2], the theory of QKD has vastly developed, with numerous protocols proposed [3,4,5,22,23,24,25,26,27,28,29,30,31,32] to improve security and increase secure-key rate (SKR). Combined with the one-time pad proved to be asymptotically safe in 1949 by Claude Shannon [1], QKD promises completely secure communication. On the other hand, QKD conventionally assumes that Eve is only limited by the laws of physics even though some assumptions might be unrealistic. For example, Eve is always assumed to have the ability to collect all photons that do not arrive at Bob’s receiver, which would make sense in cases such as fiber communication but would be too strict for wireless communication cases. Thus, interest has been rising surrounding the study of QKD with more realistic power collection assumptions and its potential applications [33,34,35,36,37,38,39,40].

In this invited paper, we present an overview of the geometrical optics restricted quantum key distribution with certain power collection restrictions applied on Eve. We start by reviewing the conventional QKD studies in Section 2 with different protocols and compare the achievable secure-key rate between the famous discrete variable protocol BB84 with decoy states added and the continuous variable Gaussian modulated QKD scheme. Then, in Section 3, we introduce the geometrical optics restricted model by limiting Eve’s collectable power with a beam splitter and showcase the lower bound results in this model. After that, we present some possible applications of this model by studying some representative scenarios with it.

## 2. Quantum Key Distribution (QKD)

With the fast development of potential applications of QKD such as quantum networks [41,42] and satellite-based quantum secure communication [43,44,45,46,47], various protocols have been proposed aimed at improved security while assuming an all-powerful eavesdropper. For example, the first QKD protocol BB84 was studied in 1984 by Charles H. Bennett et al. to use polarization states to securely distribute secret keys [2]. It was also known as the first prepare-and-measure (PM) model as it exploits the result of quantum indeterminacy that measuring an unknown quantum state in general changes the state. It was then simplified to the B92 protocol by using two non-orthogonal states [3] before extending to its entanglement-based (EB) version BBM92 [4] in 1992.

Different from the PM models, the EB models use entangled pairs in the transmission stage to distribute secret keys to the two communication parties. BBM92 was also considered as an improvement to the first EB model E91 [5], which uses three mutually unbiased bases instead of two in BBM92. There was also an important equivalence established between PM and EB models in [4] that the security proof of one implies the same for the other.

However, when it comes to device-independent (DI) studies, EB models have advantages over PM models [23] since the security proofs of DI-QKD are mainly based on the violation of Bell inequalities [48,49,50,51]. Some PM models are proven to be partially DI [52] The device independence study was first proposed in [6] using internal operations to “self-test” quantum apparatus. Different protocols have since been studied [22,23,24].

Another important category of quantum key distribution protocols is the continuous-variable (CV-) QKD. Different from most protocols described above, which are called discrete-variable (DV) protocols that rely on single photon sources and single photon detectors, CV protocols encode keys into CV observables of light fields [53]. This enabled CV protocols to be more easily implementable as it is compatible with most current communication devices. The first protocol using squeezed states [25] was proposed in 2000, which generalizes the BB84 protocol using squeezed states. In 2002, another important CV protocol GG02 using Gaussian modulated coherent states [26] was proposed as coherent states are much easier to generate experimentally. 

Other interesting directions in QKD research include using decoy states [27,28,29,54] against photon number splitting (PNS) attack [55] where the eavesdropper exploits the loophole of a non-ideal single photon transmission; finite-size analysis [56] where the transmitted sequence is not large enough for asymptotic security analysis; measurement-device-independent (MDI-) QKD [57] that comes from DI-QKD but assumes perfect preparation of the states; and high-dimensional QKD that exploits high dimensional degrees of freedom such as the orbital angular momentum (OAM) [30,31,32] and the temporal-spectral [58,59] aimed at increasing key rates, etc. Here we present introductions to two representatives in DV and CV protocols:

### 2.1. BB84

**Figure 1:**
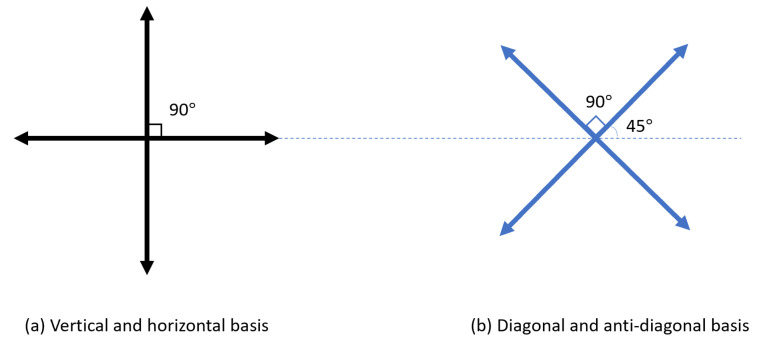
BB84 polarization bases.

BB84 protocol uses single photons to distribute secret keys. First, Alice randomly prepares a sequence chosen from two sets of orthogonal bases as in Figure 1 and sends them to Bob.

Next, Bob would also randomly choose from these two sets of orthogonal bases to measure the received photons. After completing the measurements, Bob would report his basis of measurement. If Alice’s preparing basis is the same as Bob’s measurement basis, then the result should be the same, which would be the sifted keys. 

If Eve intercepts the photons transmitted, performs a measurement of her own, and resends the photons to Bob, then when Eve’s measurement basis is not the same as Alice’s and Bob’s, the polarization state would be changed so that the sifted keys would be different on Alice’s and Bob’s side. Thus, either Alice or Bob can reveal some of the sifted keys publicly for the other party to compare and detect possible eavesdropping. An illustrative example of this process is shown in Table 1.

### 2.2. GG02

**Figure 2:**
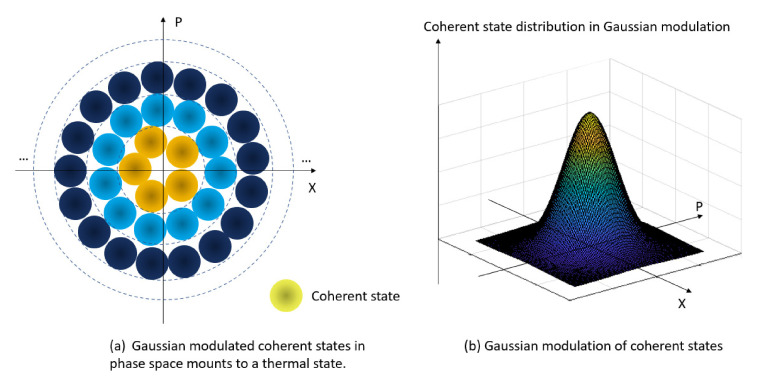
Gaussian modulated coherent states distributed on phase space.

GG02 protocol uses Gaussian modulated coherent states, as in Figure 2, to distribute secret keys. First, Alice generates random real number pairs (a_x_, a_p_) from two independent Gaussian distributions with given modulation variances and sends them to Bob. Next, Bob randomly chooses to measure either x or p quadrature components. 

After all the transmission and measurements are done, Bob discloses for each measurement whether he measured x or *p* components. Then, Alice retains the corresponding a_x_ and a_p_ values. Secret keys can then be extracted with certain reconciliation and privacy amplification. 

For these protocols, if Bob is the one performing the measurement and Alice is post-processing its outcomes to infer Bob’s encodings, assisted by classical communications from Bob to Alice, this is the reverse reconciliation scheme. Otherwise, it is the direct reconciliation scheme. Here we present a secure key rate lower bound (achievable rate) comparison between CV Gaussian modulation protocol with coherent states, heterodyne detection, reverse reconciliation, and DV protocol Decoy-State (DS-) BB84, of which detailed calculations can be found in [33,60]. We assume that a weak coherent-state source with signal-state pulses is used which transmits μ photons per pulse on average at a rate R states per second over an Alice-to-Bob channel with overall transmissivity η. Thermal noise is denoted as ne per mode.

**Figure 3:**
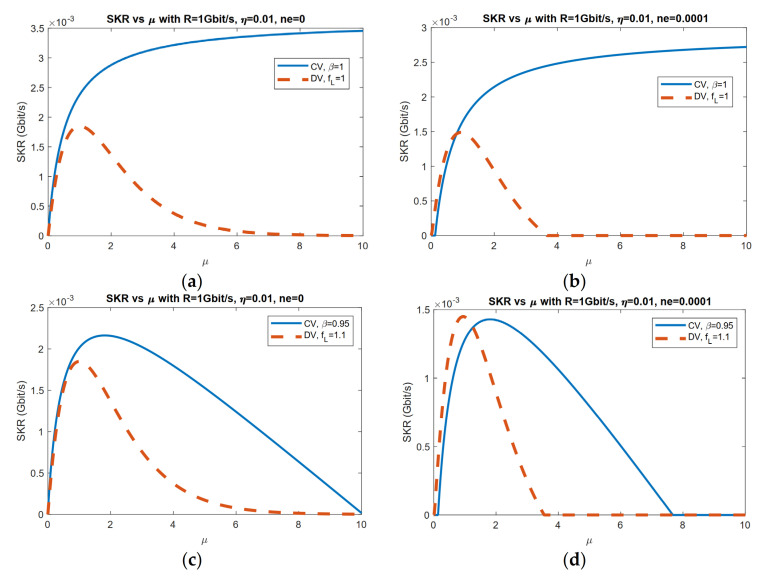
Comparison of CV Gaussian modulation protocol with coherent states, heterodyne detection, reverse reconciliation, and DV protocol DS-BB84 with mean photon number per input mode. (**a**) Perfect reconciliation in pure loss channel. (**b**) Perfect reconciliation with ne=0.0001. (**c**) Imperfect reconciliation in pure loss channel. (**d**) Imperfect reconciliation with ne=0.0001.

**Figure 4:**
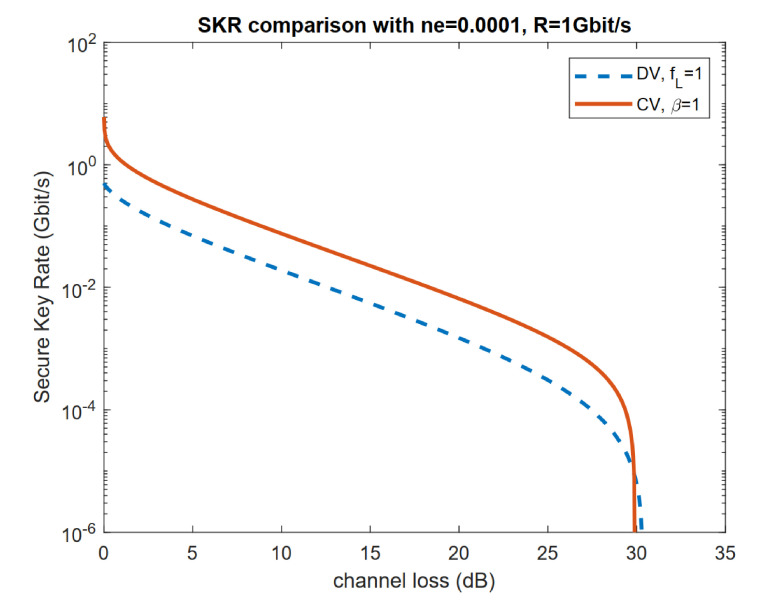
Comparison of CV Gaussian modulation protocol with coherent states, heterodyne detection, reverse reconciliation, and DV protocol DS-BB84 with channel loss. Here the input power is optimized correspondingly. Reconciliation is perfect for both CV and DV protocols.

In Figure 3a,b, the reconciliation efficiency β for CV protocol and $f_{L}$ for DV protocol are both set to one. By comparing Figure 3a,c, we can see that in a pure loss channel (ne=0), the CV protocol always outperforms its DV counterpart. However, when thermal noise is non-zero, DV can outperform CV, especially when reconciliation is not perfect. We can also compare DV and CV results with input power optimized, as in Figure 4, where the input power is optimized correspondingly with perfect reconciliation. We can see that although Gaussian-modulated CV protocol has advantages over DS-BB84 on the secure key rate, it does not outperform DS-BB84 when it comes to the transmission distance as channel loss increases with increasing transmission distance.

## 3. Geometrical Optics Restricted Model

**Figure 5:**
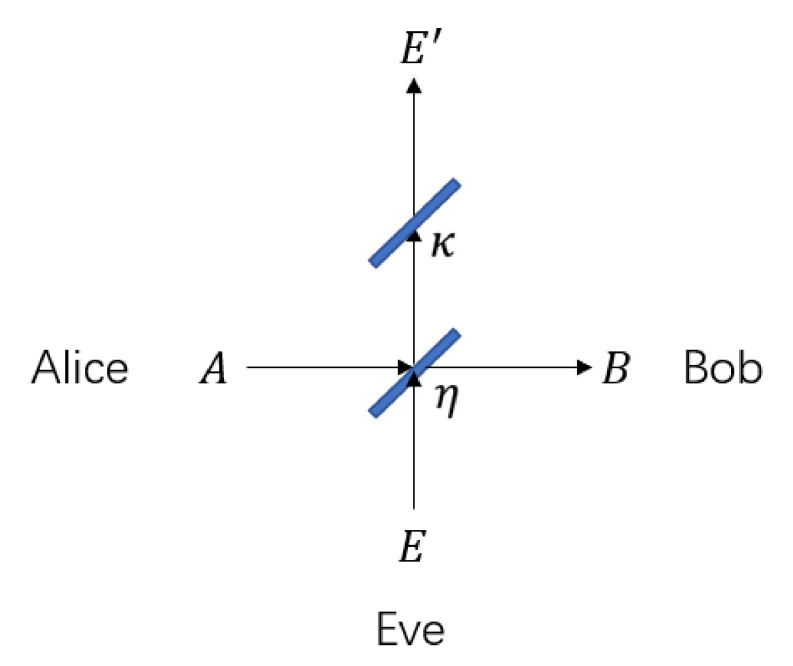
The wiretap channel notation of the geometrical optics restricted model.

In this section, we introduce the geometrical optics restricted model with realistic power collection restriction on the eavesdropper. In [33], a wiretap channel is used to denote the power collection restriction on Eve as in Figure 5. Here the beam splitter with transmissivity κ denotes that Eve can only collect κ fraction of the photons that do not arrive at Bob’s receiver. The Alice-to-Bob channel is with transmissivity η.

Similar notations have been seen in broadcast channel studies [61,62]. Starting from the Hashing inequality [63] the lower bound on the secure key rate for both direct and reverse reconciliation were derived without a specified detection scheme on one of the communication parties:

(1) $$K_{\to }\geq \beta g\left(ne\left(1-\eta \right)+\eta \mu \right)-\sum_{i}g\left(\frac{\nu_{y_{i}}^{ER}-1}{2}\right)-\beta g\left(ne\left(1-\eta \right)\right)+g\left(ne\left(1-\eta \kappa \right)\right)$$

(2) $$K_{\leftarrow }\geq \beta g\left(\mu \right)-\sum_{i}g\left(\frac{\nu_{y_{i}}^{ER}-1}{2}\right)-\beta g\left(\mu -\frac{\eta \mu \left(1+\mu \right)}{1+ne-ne\eta +\eta \mu }\right)+\sum_{i}g\left(\frac{\nu_{y_{i}}^{ER}-1}{2}\right)\;$$

(3) $$g\left(x\right)=\left(x+1\right)\log_{2}\left(x+1\right)-x\log_{2}x$$

where detailed expressions of $\nu_{y_{i}}^{ER}$ can be found in [33].

**Figure 6:**
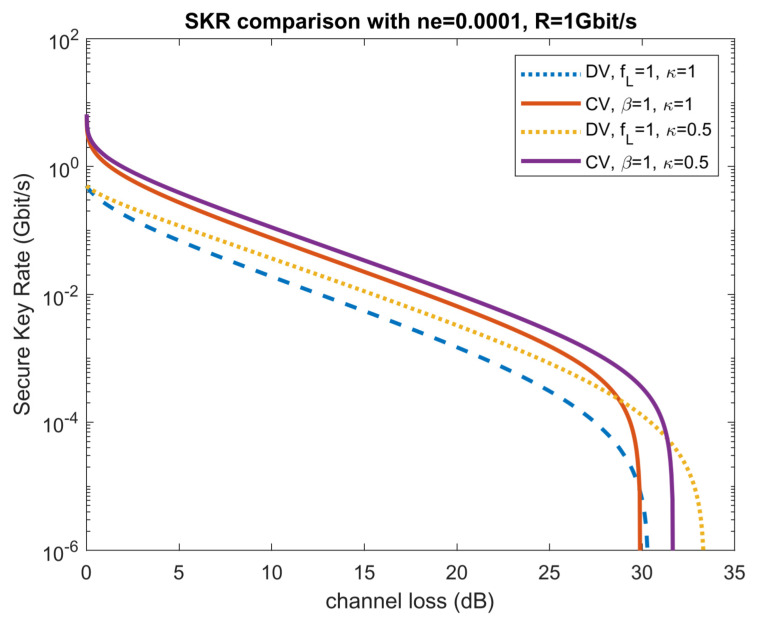
DV protocol DS-BB84 and CV Gaussian modulation protocol with coherent states, heterodyne detection, and reverse reconciliation SKR comparison with input power optimized.

Here we reproduce the comparison in Figure 4 between DV protocol DS-BB84 and CV Gaussian modulation protocol with coherent states, heterodyne detection, and reverse reconciliation as in Figure 6. We retain the results from Figure 4, as κ=1 case and plotted the DV and CV rate with κ=0.5. We can see an increase in the achievable rate in both CV and DV protocols and that the CV protocol only holds advantages over the DV protocols when channel loss is small. We can also see that when κ=0.5, the rate goes to zero at a larger channel loss, suggesting larger transmission distance in this case.

The geometrical optics restricted model has multiple potential applications in different scenarios of practical importance. Here we present some possible directions.

### 3.1. Application of Geometrical Optics Restricted Model: Limited Aperture Size Analysis

Different from the assumptions in conventional QKD study that Eve is unlimited in her ability of power collection, in most realistic application scenarios, especially in wireless communication, Eve is limited by her receiver aperture size. Taking free-space optical communication link as an example, the receiver aperture size usually ranges from a few centimeters to a few decimeters. If we only restrict Eve’s aperture size but grant her mobility of her aperture, which could be accomplished through unmanned aerial vehicle (UAV) or usage of a spy satellite during satellite communications, we can study the security of specific application occasions.

**Figure 7:**
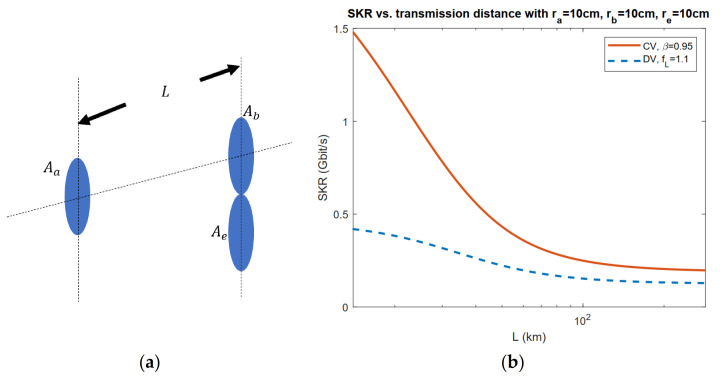
(**a**) Setup of the limited aperture scenario. $A_{a}$, $A_{b}$, $A_{e}$ respectively refer to the aperture area of Alice (radius $r_{a}$), Bob (radius $r_{b}$), and Eve (radius $r_{e}$). L is the distance between Alice’s aperture and Bob’s. (**b**) CV and DV SKR lower bounds versus transmission distance with optimized input power. Gaussian beam with beam waist $W_{0}=r_{a}$ and wavelength λ=1550 nm is transmitted. The space temperature is set to T=3 K.

In [35,37,38], the straightforward case scenario of a limited-sized aperture of Eve is considered where Eve places her aperture beside Bob’s receiver in a satellite-to-satellite communication scheme as in Figure 7a. It is shown in Figure 7b that the rate tends to be a constant when the transmission distance is sufficiently large. 

This was also derived in detail as in Equations (4) and (5), where m is the ratio of Eve’s aperture size versus Bob’s aperture size.

(4) $$\underset{\mu \to \infty ,L\to \infty }{\lim}K_{\to }\geq -\log_{2}m$$

(5) $$\underset{\mu \to \infty ,L\to \infty }{\lim}K_{\leftarrow }\geq -\log_{2}{\frac{m}{m+1}}^{1+m}e\;$$

**Figure 8:**
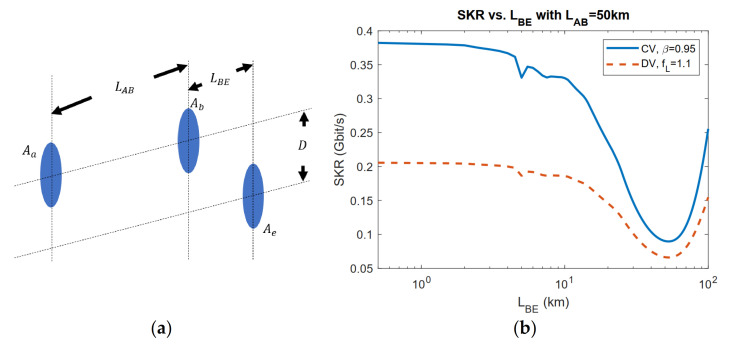
(**a**) Setup of the dynamic positioning of Eve. $A_{a}$, $A_{b}$, $A_{Eve}$ respectively refer to the aperture area of Alice (radius $r_{a}$), Bob (radius $r_{b}$), and Eve (radius $r_{e}$). $L_{AB}$ is the distance between Alice’s aperture and Bob’s. $L_{BE}$ is the distance between Bob’s aperture and Eve’s. (**b**) CV and DV lower bound secret keys versus Bob-to-Eve distance $L_{BE}$ with Alice-to-Bob distance $L_{AB}=50\;k$m. Gaussian beam with beam waist $W_{0}=r_{a}=r_{b}=r_{e}=10\;c$m and wavelength λ=1550 nm is transmitted. The space temperature is set to T=3 K.

In [36,40], the case with dynamically positioned eavesdropper aperture is considered with Eve’s position being optimized, as in Figure 8a. In Figure 8b, both CV and DV lower bounds are presented with optimized Eve’s position. Assuming the Gaussian beam is transmitted, because of the cylindrical symmetry of a Gaussian beam, the distance D between Eve’s aperture to the beam transmission axis can be used to denote Eve’s position combined with Bob-to-Eve distance $L_{BE}$. It is clear that by optimizing Eve’s position, advantages over Alice and Bob can be further obtained by Eve compared with Figure 7b.

### 3.2. Application of Geometrical Optics Restricted Model: Exclusion Zone Analysis

**Figure 9:**
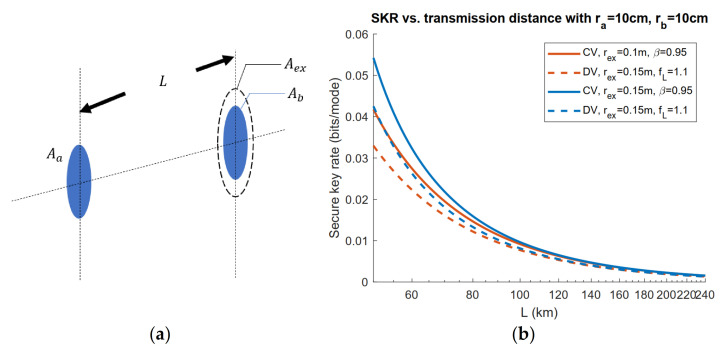
(**a**) Setup of exclusion zone scenario. $A_{a}$, $A_{b}$, $A_{ex}$ respectively refer to the area of Alice’s aperture (radius $r_{a}$), Bob’s aperture (radius $r_{b}$), and the exclusion zone (radius $r_{ex}$). L is the distance between Alice’s aperture and Bob’s. (**b**) CV and DV lower bound of secret keys versus transmission distance L with or without an exclusion zone. Gaussian beam with beam waist $W_{0}=r_{a}$ and wavelength λ=1550 nm is transmitted. The space temperature is set to T=3 K.

From the defense point of view, one of the most effective ways to suppress Eve’s power collection ability is to set an exclusion zone around the legitimate receiver. In [39] an exclusion zone is assumed to be set surrounding the legitimate receiver, excluding the eavesdropper Eve from collecting photons in this region, as in Figure 9a. In Figure 9b, an exclusion zone is shown to increase the secure key rate for both CV and DV protocols, but this is more effective when the transmission distance is not too large.

## 4. Discussion

In this paper, we provided a brief overview of the geometrical optics restricted QKD and discussed its potential applications. We started by reviewing some of the existing QKD schemes before going into the geometrical optics restricted model notation in a wiretap channel that can better characterize the power collection state of some realistic scenarios instead of attributing too much power to Eve. After we introduced the lower bound results in this model, we then presented some of the application directions of this model, mostly in free-space channels such as satellite communication. We showcased selected results from both Eve’s side with her optimized position strategy and the communication parties’ side with an exclusion zone as a defense strategy.

## Figures and Tables

**Table 1:** BB84 protocol process illustration.

Random Bits	0	1	0	0	1	1	1	0	1
Alice basis	a	a	b	a	b	a	b	b	a
Polarization state sent	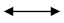	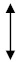	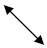	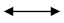	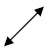	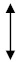	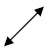	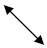	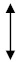
Bob basis	b	a	a	b	b	a	b	a	b
Bob measurement results	random	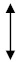	random	random	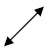	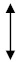	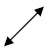	random	random
Sifted keys		1			1	1	1		
